# Low-Cost Air Quality Measurement System Based on Electrochemical and PM Sensors with Cloud Connection

**DOI:** 10.3390/s21186228

**Published:** 2021-09-16

**Authors:** Patricia Arroyo, Jaime Gómez-Suárez, José Ignacio Suárez, Jesús Lozano

**Affiliations:** Industrial Engineering School, University of Extremadura, 06006 Badajoz, Spain; parroyoz@unex.es (P.A.); jaimegs@unex.es (J.G.-S.); jmarcelo@unex.es (J.I.S.)

**Keywords:** air quality, low-cost, calibration, electrochemical gas sensors, portable device

## Abstract

This paper presents a portable device for outdoor air quality measurement that provides concentration values for the main pollutants: NO_2_, NO, CO, O_3_, PM_2.5_ and PM_10_, and other values such as temperature, humidity, location, and date. The device is based on the use of commercial electrochemical gas and optical particle matter sensors with a careful design of the electronics for reducing the electrical noise and increasing the accuracy of the measurements. The result is a low-cost system with IoT technology that connects to the Internet through a GSM module and sends all real-time data to a cloud platform with storage and computational potential. Two identical devices were fabricated and installed on a mobile reference measurement unit and deployed in Badajoz, Spain. The results of a two-month field campaign are presented and published. Data obtained from these measurements were calibrated using linear regression and neural network techniques. Good performance has been achieved for both gaseous pollutants (with a Pearson correlation coefficient of up to 0.97) and PM sensors.

## 1. Introduction

Ambient air quality is an issue of continuous concern from the point of view of human health due to the many factors that cause serious diseases. Environmental pollution is the cause of certain neurological disorders [1] and certain respiratory problems aggravated by the presence of elements such as NO_x_ [2]. Some mental diseases are associated with the presence of ozone [3] and particulate matter (PM) is often the cause of lung cancer [4]. The World Health Organization (WHO) estimated in 2012 that one out of nine deaths worldwide were related to air pollution and, of these deaths, about three million were related to ambient (outdoor) air pollution [5]. Furthermore, WHO also indicates that ozone and PM are responsible for increased risk of respiratory mortality and morbidity, while NO_x_, ozone and PM are responsible for allergic reactions.

Pollutant emissions are closely linked to human social and economic activities as demonstrated by the mobility restrictions during the COVID-19 pandemic. According to a recent study [6], in the US, NO_2_ and particulate matter (PM_2.5_) concentrations were drastically reduced during confinement (by more than 25% for NO_2_). In China, reductions in NO_x_ emissions of at least 15% were detected by satellite observations, with the largest reductions (up to 50%) occurring in large cities. After lockdown, a mild recovery of pollution levels was observed [7].

The main contributors to air pollution are often emissions from transportation, whether by land [8], air [9] or sea [10], and industrial development, especially in megacities [11,12].

The traditional approach to air quality monitoring is based on the use of conventional measurement stations based on very expensive, reliable and complex sensor systems located at only a few fixed sites, due to the high cost of the equipment. Moreover, their use is economically prohibitive for locating the most important pollution hotspots in a region and capturing their temporospatial heterogeneity. On the other hand, there are also mobile stations, based on the same technology as fixed stations (and therefore expensive), to measure where fixed stations cannot reach. Their number is even smaller than that of fixed stations, due to their high operational cost, as they require personnel and transport by automobiles (cars or vans) which, in most cases, are not very environmentally friendly. Nevertheless, the number of fixed and mobile stations is usually not sufficient to obtain air quality maps with a high level of resolution in real-time.

As a complement to reference (conventional) measurement stations, low-cost sensor devices have emerged in recent years that, because they can be distributed ubiquitously, allow for more detailed mapping of areas where conventional monitoring stations cannot reach [13]. In addition, owing to the use of wireless sensor networks (WSNs), embedded GPS systems and other communication technologies, real-time, location-indicated ambient air pollution data can be obtained [14]. In this context, the European project NanoSen-AQM [15] was established, which seeks to develop gas sensors and low-cost systems to measure and display air quality in real-time, promoting the monitoring of air quality in a massive, distributed and ubiquitous way.

Within low-cost air quality measurement systems, the main element lies in the operation of gas sensors. A large number of studies with low-cost sensors have been carried out for this purpose, which are reported in review studies [16,17]. As some authors outline, the main technologies used are metal-oxide (MOX) sensors, electrochemical sensors, nondispersive infrared (NDIR) sensors and photoionization detectors (PID) [18]. In systems that are not intended for personal use (although portable), electrochemical sensors perform well in detecting pollution. It is a selective technology and, although not the most inexpensive, it is considered low-cost compared to reference systems. In general, studies suggest that these sensors perform best in the laboratory, where conditions are controlled [19,20]. Therefore, field calibration is required, and this is where the different techniques for this purpose play a role. In the current literature dealing with electrochemical sensors, the most commonly used techniques are linear regression [20,21,22], artificial neural networks [23], random forest trees and hybrids [24,25].

Furthermore, it is important to consider the exponential growth of research in the field of the Internet of Things (IoT) since 2010 [26]. This technology makes it possible to interconnect sensor elements with the Internet. On the other hand, cloud computing has also been an emerging technology in recent years. In this case, systems can be provided with virtually unlimited storage and high processing capacity. In this way, the integration of IoT and cloud computing with air quality measurement systems makes it possible to work with a large number of sensors in real-time. That integration is already beginning to appear in recent research [27,28,29,30]. The system presented in this paper has benefited from these technologies by making use of a platform (developed in the NanoSen-AQM project) that stores and processes air quality data, and makes it accessible to the public.

The contribution of this work is the development of a low-cost air monitoring device that measures the main pollutants present in the air (CO, NO_2_, NO, O_3_, PM_10_, PM_2.5_) and temperature and relative humidity, with a specific design of the electronic instrumentation for reducing the noise and increasing the accuracy of the measurements. The fabrication of two identical prototypes for field validation in parallel with a certified reference station are located in a hotspot traffic site, its calibration using different techniques and the results of a first measurement campaign. This extends the range of real-world studies with the Alphasense A4 series, as most studies with Alphasense sensors include the B4 series.

The remainder of the paper is divided into three main parts. Section 2 describes the system that was developed, the sensors used, the data acquisition method and the different calibration procedures applied. Section 3 presents and discusses the results obtained in the field campaign. Finally, Section 4 summarizes the main conclusions.

## 2. Materials and Methods

### 2.1. Sensor Device

A device was designed to estimate air quality in both mobile and fixed stations. It is intended for measuring the concentration of gaseous pollutants (NO_2_, NO, CO and O_3_), together with PM_10_ and PM_2.5_ particles. Among its main features are communication by GSM with the developed cloud, GPS geolocation, Bluetooth control and SD memory storage.

The gas sensors are four-electrode electrochemical sensors, A4 series, supplied by Alphasense (Essex, UK). The gas sensors are housed in a metal enclosure to reduce the effects of electromagnetic interference. An external 24-bit digital-to-analogue converter with eight inputs (ADS1256IDBT, Texas Instruments, Dallas, TX, USA) is used to read the value of these sensors. The particle sensor selected for PM_10_ and PM_2.5_ detections is OPC-N3 (also from Alphasense). It uses laser beams to detect particles from 0.35 to 40 µm in diameter with 24 bins. Count measurements are converted into mass concentrations of PM_1.0_, PM_2.5_ and PM_10_ using embedded algorithms. These sensors have demonstrated good performance in other air quality detection works [31,32,33,34,35]. The SHT21 humidity and temperature sensor is also incorporated, as these could help in the interpretation of the results, and by providing extra information about the environment. The microcontroller used for the control of the complete system is the STM32L476 model from ST (STMicroelectronics, Plan-les-Ouates, Switzerland). It is an ultra-low-power microcontroller based on a high-performance 32-bit Arm Cortex-M4 core operating at a frequency of up to 80 MHz. For Bluetooth communication, mainly used for debugging and operational control purposes, a low energy module (RN4871, Microchip, Chandler, AZ, USA) was incorporated. A GSM module SIM808 (SIMCom, Shanghai, China) and an Ethernet module W5500 (WIZnet, Seongnam-si, Korea) are used for data transmission. In addition, the SIM808 module incorporates GPS to determine the location of each device. It also provides local storage of data using a microSD card that can be inserted directly into the main control board.

**Figure 1:**
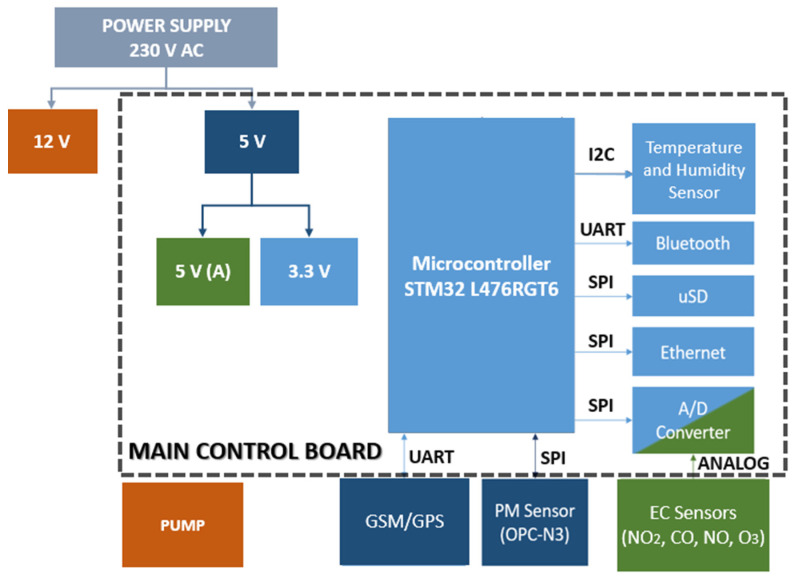
General diagram of the electronic design.

A model ECL15UT02-S (XP power, Singapore) is used to supply power for the entire equipment: 100–240 VAC, 0.6 A, 50–60 Hz. The voltage is converted to 12 and 5 VDC; 12 V is used to power the pump (1410VD/1.5/E/BLDC model from Thomas, Fürstenfeldbruck, Germany) that drives the air to the gas sensors and the 5 VDC supplies the GPS module and the PM sensor. In addition, these 5 VDC are converted to 3.3 VDC for the power supply for most of the electronic components, including the microcontroller and the Bluetooth module. Finally, the power supply for the electrochemical sensors is isolated through a ferrite core to avoid adverse interference derived from the digital signals. The general diagram of the equipment, specifying operating voltages and communication buses for each component, is shown in Figure 1.

**Figure 2:**
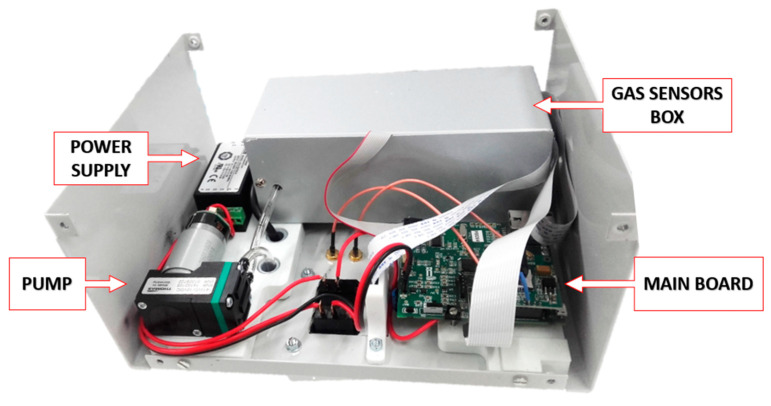
Main parts of the designed system.

The general operation of the system is described below. The data from the gas sensors, particle sensor, temperature, humidity and positioning are collected by the microcontroller. This microcontroller packages the data and sends it to the NanoSen-AQM server using the GSM or Ethernet module through the MQTT communication protocol. In addition, these data are also stored on an SD card. Through Bluetooth communication, it can be checked that the device is working properly, through a debug monitoring function. Figure 2 shows a picture of the designed system (uncovered) with some of the main parts labeled.

**Figure 3:**
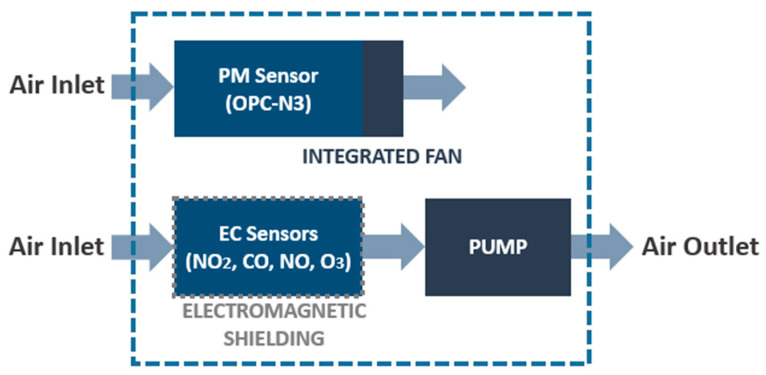
Airflow diagram.

The air is sampled through two different inlets. First, the OPC (particulate matter sensor) collects the air through its own sampling system using a fan. In addition, there is another air inlet for the gas sensor cell. The air passes through the gas sensors, included in an electromagnetic shield, and is expelled outside by a pump. Figure 3 shows an airflow diagram.

The systems include a metal cover with insulation for weather protection. This cover includes a chimney for the PM air inlet and a fastening system specifically designed for vertical or horizontal bar installation.

### 2.2. Gas Sensors

As specified above, electrochemical gas sensors are used. This type of sensor is based on an amperometric operating principle [36], which usually incorporate three electrodes: working (WE), counter electrode and reference electrode. The WE is the sensing electrode, where half of the redox reaction of the target gas takes place. The redox reaction is completed at the counter electrode. Finally, the reference electrode is used to maintain a stable WE potential. In addition, selected models incorporate a fourth auxiliary electrode (AE) to compensate for temperature dependence [37]. This electrode is identical to the WE, but is not in contact with the gas, therefore it will only be reactive to physical changes in the environment, such as temperature. In this way, the manufacturer aims to improve the correction of interferences that may be derived from the environmental conditions to which the sensor is exposed and which may have a negative effect on its operation (changes in temperature, humidity or pressure). Therefore, under ideal conditions, the subtraction of the signal from these two electrodes would result in a signal proportional to the concentration of the target gas. However, it was found that this principle of operation does not work correctly over a wide range of temperatures. For example, in some cases, negative errors are obtained with increasing temperature. In order to rectify this effect, Alphasense provides some guidance on the correction of the zero background current due to temperature within the range of −30 °C to +50 °C [38]. This algorithm is detailed in Section 2.6.1.

### 2.3. Data Acquisition

In the electrochemical sensors, the data received in mV from each of the two electrodes (auxiliary and working) are translated into the pollutant concentration in ppb. This is done using an algorithm provided by the manufacturer, including a temperature correction. Then, a change of units to µg/m^3^ for all data is performed. In the case of the PM sensor, it measures the particle number concentration directly in 24 size ranges from 0.35 to 40 μm. From these values, it calculates the mass concentration through an onboard factory calibration for PM_1_, PM_2.5_ and PM_10_, so no pretreatment is needed. Finally, the values of temperature (°C) and humidity (%) supplied by the SHT21 sensor are calculated using the equations provided by the manufacturer for this purpose. Measurements are taken each 3 s and sent to the cloud after 50 s. All data taken are 10 min and 1 h averaged and sent to the server, synchronized in time with the measurements taken with the reference station, which provides the 10 min and 1 h data.

The server is a platform developed within the framework of the NanoSen-AQM project by the University of Coimbra and the University of Evora [39,40]. This platform allows the user to have public access to air quality data via a web browser or mobile application [41,42]. Accounts can be created on the site, allowing specific clusters to be marked as favorites and alerts to be set for cases where air quality data exceeds a certain threshold. In addition, special users with “sensor owner” privileges can manage their sensors, send data from them and upload adjustment or calibration functions (in Python). The latter is the case for the devices presented in this paper. From each device, the data sent to the cloud are NO_2_ (µg/m^3^), O_3_ (µg/m^3^), NO (µg/m^3^), CO (µg/m^3^), PM_2.5_ (µg/m^3^), PM_10_ (µg/m^3^), temperature (°C), humidity (%), latitude, longitude, date and time. However, the data are also stored locally every 50 s on a microSD card. In addition to the abovementioned data, the raw values of the individual electrodes of the sensors are saved in the memory. In this way, subsequent calibrations can be performed based on these data.

### 2.4. Reference Methods

The reference station used for validation belongs to the Extremadura Air Quality Protection and Research Network (REPICA) of the Department for Ecological Transition and Sustainability of the Regional Government of Extremadura. The equipment of the reference station follows:

− O_3_: THERMO 49i-B3ZAA (UV absorption);
− NO_x_: THERMO 42i-BZMTPAA (chemiluminiscence);
− PM: DIGITEL DHA-80 (high volume sampler + gravimetric analysis). GRIMM 180 (optical laser light aerosol spectrometers) nonofficial data.

### 2.5. Field Measurement Campaign

**Figure 4:**
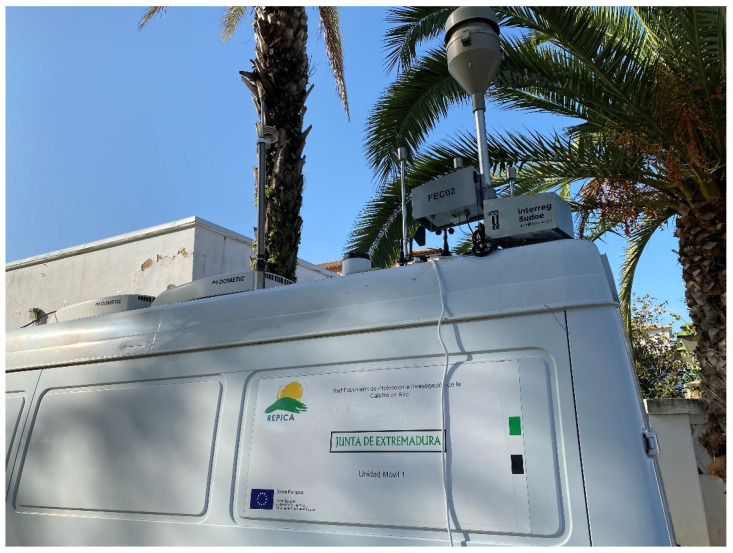
FEC01 and FEC02 installed in the reference mobile unit in Badajoz.

The experimental campaign was carried out in Badajoz (Spain) between 12 March and 17 May 2021. The prototype developed (sensor devices FEC01 and FEC02) were placed on a mobile reference unit, anchored on a pole located at the top (Figure 4) in parallel with the reference equipment. The location of the whole system was in a traffic hotspot in a central avenue of Badajoz (38°52′15″ N, 06°58′44″ W), in order to be able to measure high pollution levels. It is important to compare two identical devices with the reference system, as recommended in international regulations such as ISO 13752:1998. All the data (both low-cost sensor and reference measurements) obtained are published as Supplementary Materials for other users, which can be used to check other calibration and prediction algorithms.

### 2.6. Calibration Procedure

The data obtained in the field campaigns (see Section 2.5) was used for both calibration and validation against reference systems using different methods for comparison. In the calibration processes described (except for the one proposed by Alphasense, which was implemented in the software integrated in the system), the data from the first week were used to perform the calibration. Subsequently, the algorithm was applied to the remaining data of the campaign to study its performance. It should be noted that the data used in the studies are hourly averages, as these are the ones officially used. Python 3.8 software was used to carry out the full calibration process.

#### 2.6.1. Manufacturer Algorithm

Electrochemical amperometric gas sensors generate a background current (zero background current) in addition to the oxidation or reduction current of the sampled gas. In addition, the AE also generates a current that mainly follows the WE current. These currents can be significant and can frustrate attempts to make measurements at low gas concentrations.

The algorithms suggested by Alphasense, hereafter MA, are focused on a primary correction of this zero background current for temperature effects. A full correction is complex and secondary corrections are usually required to further correct for residual deviations and gain changes.

The proposed correction consists first of subtracting the zero offsets (parameters supplied individually for each sensor) from the electrode values. Next, a temperature-dependent compensation factor must be applied. Subsequently, the result is divided by the sensitivity parameter (also supplied by the manufacturer) to translate the result from mV to ppb. In addition, for the case of the ozone sensor (OX-A4), a NO_2_ correction must be made, since it reacts to both gases. Specifically:

$$\left[\mathrm{Pollutant}\right]\left(\mathrm{ppb}\right)=\frac{\left(S_{\mathrm{WE}}-S_{\mathrm{WE},0}\right)-n\left(S_{\mathrm{AE}}-S_{\mathrm{AE},0}\right)}{s}$$

where $S_{\mathrm{WE}}$ and $S_{\mathrm{AE}}$ are the working electrode and auxiliary electrode values, respectively; $S_{\mathrm{WE},0}$ and $S_{\mathrm{AE},0}$ are the zero offset values of the electrodes; n is a temperature-dependent parameter given by the manufacturer and s is the sensitivity constant.

After applying this algorithm, issues such as gas concentrations being negative, or appearing to be much smaller or larger than the reference values are likely to emerge. However, in general, the sensor output follows the trend of the reference gas concentration. It is due to the fact that this calibration is calculated from measurements in a laboratory environment: controlled temperatures, with dry gases, and without the presence of other gases. Several authors who have already used sensors from this manufacturer have encountered this challenge [20,43]. Based on research findings, it is more appropriate to calculate empirical correction factors: minimize the offset error so that the sensor gas reading matches as closely as possible a set of reference gas values over a period of time, e.g., one week. In this work, since the problems described above arose, it was decided to perform a one-week field calibration. Subsequently, different algorithms were applied with the purpose of improving the performance of the system.

#### 2.6.2. Single Linear Regression

Initially, a simple linear regression (SLR) of the data in µg/m^3^ obtained through the manufacturer’s algorithm was attempted to correct the data. Specifically, a double correction was made for the slope and the offset of the first week’s data with respect to the values obtained at the reference station, namely, both the slope and the offset of the dataset were corrected using the first week of data, and then recalibrated using the same process.

#### 2.6.3. Multilinear Regression

Next, a multilinear regression (MLR) based on the Mijling work [20] was performed using the raw data. In this case, a linear combination of the values of the eight electrodes (mV) from the gas sensors and the temperature and humidity from the SHT21 was proposed. For PM_10_ and PM_2.5_, the values used in the combination are, apart from the PM output in µg/m^3^, temperature, humidity and sampling flow rate (SFR) recorded by the OPC-N3.

Specifically, for the gaseous pollutant:

$$\left[\mathrm{Gaseous}\;\mathrm{Pollutant}\right]=\alpha_{0}+\overset{10}{\underset{1}{\sum }}\alpha_{i}S_{i}$$

where $\alpha_{i}$ are the regression coefficients and $S_{i}$ are the values of the eight electrodes from the gas sensors and the temperature and relative humidity from SHT21.

Lastly, for the particulate matter:

$$\left[\mathrm{PM}\right]=\alpha_{0}+\alpha_{1}{\mathrm{PM}}_{1}+\alpha_{2}{\mathrm{PM}}_{2.5}+\alpha_{3}{\mathrm{PM}}_{10}+\alpha_{4}T+\alpha_{5}\mathrm{RH}+\alpha_{6}\mathrm{SFR}$$

#### 2.6.4. Multilayer Perceptron Regressor

An artificial neural network, specifically a multilayer perceptron (MLP), was trained using the first week of data as the training set and the rest of the data as the testing set. Before applying the MLP, a normalization from −1 to 1 was applied in the case of the gas sensor values, whereas for the particulate sensor values, the data were standardized calculating the z-score. In both cases, the training and the test set were normalized/standardized in a single step. The input layer had 10 neurons (eight electrodes, temperature and humidity) in the case of gaseous pollutants and 6 neurons (3 p.m. temperature, humidity and SFR) in the case of particulate matter. The network was formed by two hidden layers and two nodes on each layer. A rectified linear unit function was used as the activation function. This model optimized the squared-loss using stochastic gradient descent with a tolerance of 0.0001. Finally, there was only one neuron on the output layer.

A summary of the algorithms and corrections applied to the low-cost sensors is presented in Table 1.

## 3. Results and Discussion

The results obtained in the field measurements carried out in Badajoz with two devices (named FEC01 and FEC02) during two months are presented and discussed.

### Model Performance

With the aim of studying the success rate for each strategy used to perform the calibration tasks, statistical indices of performance were calculated. The Pearson correlation coefficient (R^2^), the mean absolute error (MAE), the mean squared error (MSE) and the coefficient of determination (r^2^) are presented in Table 2.

From these results, it can be deduced that the performance of the system with laboratory calibration is unreliable, often giving negative concentration values. Even attempting to correct the data with linear regression, no improvement is achieved. This has two main reasons: first, our simple linear regression model uses concentration values, which are already biased by the manufacturer’s algorithm. Second, using SLR it is possible to fix the slope and the offset to 1 and 0, respectively, but this method does not improve the deviation of the data, thereby providing low statistical indices.

However, using the raw data obtained from each sensor together with temperature and humidity as input, more acceptable output is achieved. At best, an R^2^ of 0.95 is achieved by using the MLP technique to calibrate the O_3_ data recorded by the FEC01 device.

**Figure 5:**
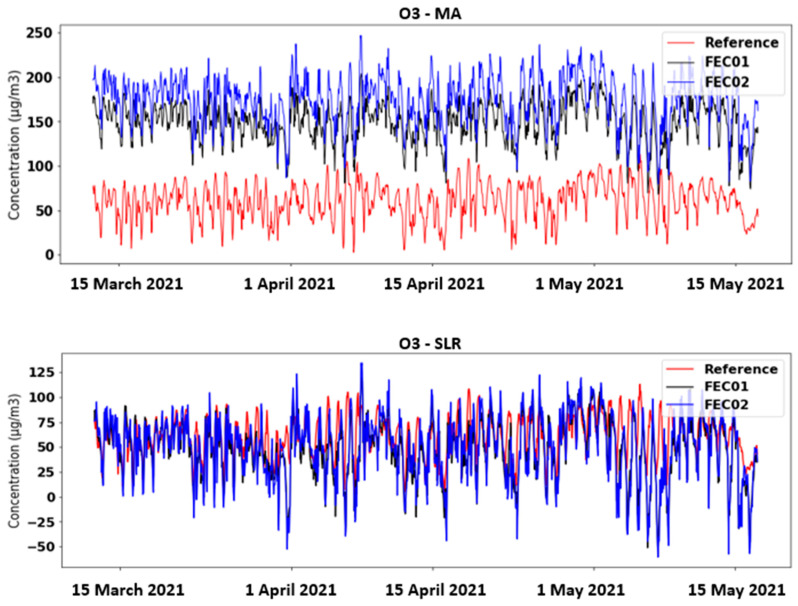
Time chart of O_3_ concentrations obtained with MA and SLR calibration techniques.

**Figure 6:**
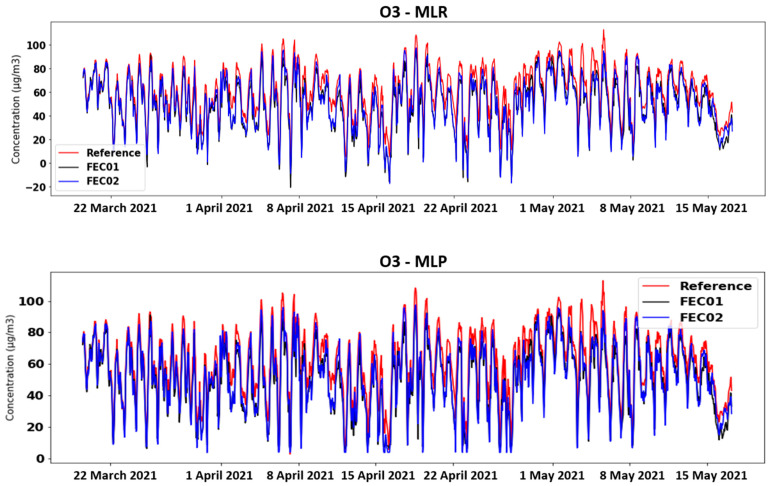
Time chart of O_3_ concentrations obtained with MLR and MLP calibration techniques.

Figure 5 shows the O_3_ time plots of FEC01, FEC02 and the reference station in the MA and SLR calibration cases. In the first case, it can be appreciated that the two devices register much higher values than the actual ones, although the trend seems similar to the reference value. Attempting to correct these data with SLR, it is possible to achieve levels closer to the reference. However, there is still an upper and lower offset in several areas and even negative concentration values are obtained (which is impossible). These plots are significantly improved when applying the MLR and MLP techniques with the raw data as input (Figure 6). Nevertheless, in the first case, negative values are still obtained, but less frequently than above. This effect is fully corrected by applying neural networks (MLP), although a negative offset is observed in some areas.

**Figure 7:**
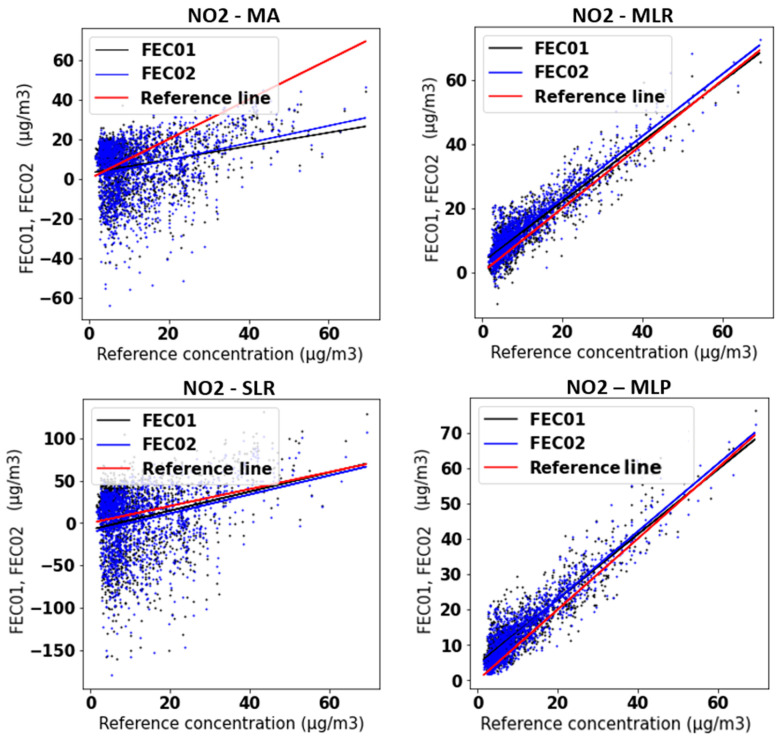
FEC01 and FEC02 concentrations obtained with MA, SLR, MLR and MLP vs. reference concentration.

Figure 7 depicts the four NO_2_ concentrations obtained from the FEC01 and FEC02 devices against those obtained from the reference station. It can be noticed that when SLR is applied to the MA data, the slope is corrected. However, the large scatter already existing in the data is multiplied. Conversely, by applying the MLR and MLP techniques, apart from correcting the slope, the data scattering also decreases notably.

**Figure 8:**
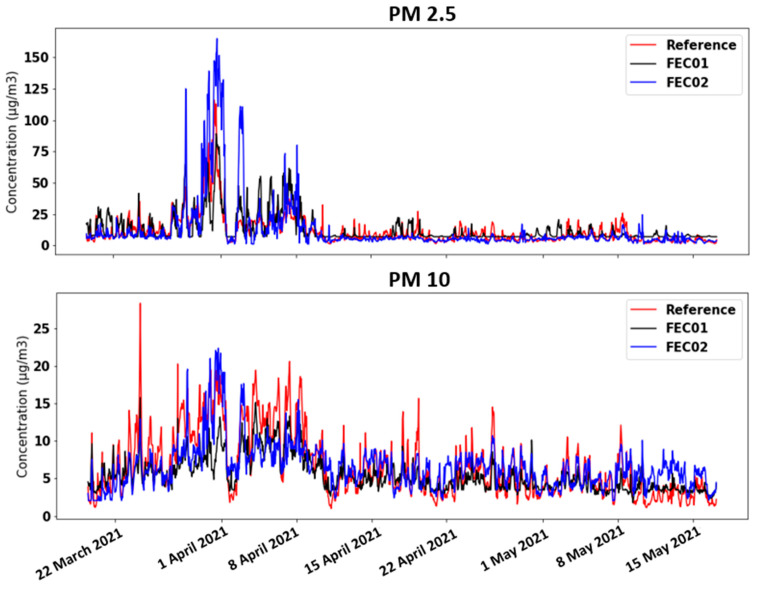
Time chart of PM_10_ and PM_2.5_ concentrations obtained with MLP calibration technique.

Regarding PM_10_ and PM_2.5_ data, as shown in Table 2, good performance levels are not achieved. During certain hours or days, the FEC01 and FEC02 devices reach much higher PM values than the reference station (Figure 8). It can be evidenced in the case of PM_2.5_ with FEC02 around 1 April 2021. These random effects, as can be appreciated, were not corrected by the calibrations. It was initially thought that this could be linked to the interference that high humidity can have on the optical particle sensors, or even rainfall. However, we studied the rainfall time series provided by AEMET for the same time period and location, and also the relative humidity data from the low-cost sensors and the reference station, and no correlation between rainfall or humidity and high particle values was found.

## 4. Conclusions

A home-designed and home-developed electronic system was developed for the measurement of the main pollutants responsible for air quality. Measurement campaigns were carried out with two identical prototypes in parallel with reference methods to study their performance. The data obtained are shared as Supplementary Materials for the scientific community. The electrochemical sensors implemented, using the factory algorithm, provide concentration values that are very different from the reference values. However, the trend of these signals (prototypes and reference) does appear to be similar. Different regression and neural network techniques were implemented with the aim of refining these values. The raw values (working and auxiliary electrode voltage) of each gas sensor were used as inputs for the network. In this way, the R^2^ was improved from 0.07–0.47 to 0.83–0.95.

However, for the particulate matter values, only the R^2^ values were improved to 0.78 in the case of PM_10_. It was observed that the OPC-N3 sensor reports much higher values than the reference on certain dates. Moreover, it was ruled out that this error is due to rainfall and it could be due to fog or other interferences, which could be compensated by software. In future work and campaigns, this effect will be studied.

Calibrations presented in this paper are preliminary estimations, used in order to study the potential good performance of the devices that were developed. In the future, these calibrations will be improved to optimize the complete system. Data from field campaigns at other locations and stations will be used for this purpose. In addition, confidence limit studies based on the European Validation Guide for air quality sensors will be applied.

To conclude, low-cost systems can complement the air quality monitoring networks of competent institutions, but good electronic design is important to obtain the best values from the sensors without electromagnetic interference. On the other hand, field validation is essential for adjusting calibration parameters under actual operating conditions and to obtain more successful prediction of concentrations and less uncertainty in measurement.

## Figures and Tables

**Table 1:** Summary of calibration algorithms applied to the air quality data from low-cost sensors.

Technique	Inputs	Outputs
Manufacturer Algorithm (laboratory calibration) (MA)	NO_2__WE (mV), NO_2__AE (mV), T (°C)	NO_2_ (µg/m^3^)
	NO_WE (mV), NO_AE (mV), T (°C)	NO (µg/m^3^)
	CO_WE (mV), CO_AE (mV), T (°C)	CO (µg/m^3^)
	O_3__WE (mV), O_3__AE (mV), T (°C)	O_3_ (µg/m^3^)
	-	PM_10_ (µg/m^3^), PM_2.5_ (µg/m^3^)
Simple Linear Regression (SLR)	NO_2_ (µg/m^3^)	NO_2_ (µg/m^3^)
	NO (µg/m^3^)	NO (µg/m^3^)
	CO (µg/m^3^)	CO (µg/m^3^)
	O_3_ (µg/m^3^)	O_3_ (µg/m^3^)
	PM_10_ (µg/m^3^)	PM_10_ (µg/m^3^)
	PM_2.5_ (µg/m^3^)	PM_2.5_ (µg/m^3^)
Multiple Linear Regression (MLR)	NO_2__WE (mV), NO_2__AE (mV), NO_WE (mV), NO_AE (mV), CO_WE (mV), CO_AE (mV), O_3__WE (mV), O_3__AE (mV), T (°C), RH (%)	NO_2_ (µg/m^3^)
		NO (µg/m^3^)
		CO (µg/m^3^)
		O_3_ (µg/m^3^)
	PM_1_ (µg/m^3^), PM_2.5_ (µg/m^3^), PM_10_ (µg/m^3^), T (°C), RH (%), SFR	PM_10_ (µg/m^3^)
		PM_2.5_ (µg/m^3^)
Multilayer Perceptron (MLP)	NO_2__WE (mV), NO_2__AE (mV), NO_WE (mV), NO_AE (mV), CO_WE (mV), CO_AE (mV), O_3__WE (mV), O_3__AE (mV), T (°C), RH (%)	NO_2_ (µg/m^3^)
		NO (µg/m^3^)
		CO (µg/m^3^)
		O_3_ (µg/m^3^)
	PM_1_ (µg/m^3^), PM_2.5_ (µg/m^3^), PM_10_ (µg/m^3^), T (°C), RH (%), SFR	PM_10_ (µg/m^3^)
		PM_2.5_ (µg/m^3^)

**Table 2:** Performance statistical indices for the calibration techniques implemented.

	Pollutants	R^2^				MAE				MSE				R^2^			
		MA	SLR	MLR	MLP	MA	SLR	MLR	MLP	MA	SLR	MLR	MLP	MA	SLR	MLR	MLP
**FEC01**	**NO_2_**	0.07	0.07	0.81	0.83	11.89	35.59	0.94	3.65	230.13	1784.15	26.34	23.80	−1.24	−16.40	0.73	0.75
	**NO**	0.22	0.22	0.85	0.92	28.41	10.17	2.06	1.25	869.89	149.22	7.52	3.60	−15.79	−1.88	0.84	0.92
	**CO**	0.47	0.47	0.38	-	60.79	0.06	45.36	-	5125.31	0.01	3211.12	-	−0.21	−0.51	0.17	-
	**O_3_**	0.34	0.34	0.94	0.95	87.11	17.30	10.86	9.22	7981.32	724.46	145.11	106.02	−18.24	−0.75	0.66	0.75
	**PM_10_**	0.66	0.66	0.70	0.78	18.16	3.14	2.38	1.72	443.78	22.73	9.92	5.36	−24.66	−0.31	0.47	0.61
	**PM_2.5_**	0.27	0.27	0.45	0.50	9.50	111.40	8.10	5.93	147.95	23.3 × 10^3^	117.56	85.19	−0.06	−166.01	0.20	0.42
**FEC02**	**NO_2_**	0.09	0.08	0.85	0.86	12.34	27.94	4.11	3.81	266.46	1423.04	27.21	24.09	−1.60	−12.88	0.72	0.75
	**NO**	0.41	0.41	0.88	0.89	6.91	9.14	2.09	1.76	76.90	117.12	7.59	6.40	−0.48	−1.25	0.84	0.86
	**CO**	0.45	0.45	0.38	-	0.07	0.07	0.05	-	0.01	0.01	0.00	-	−0.67	−0.89	0.16	-
	**O_3_**	0.22	0.21	0.93	0.94	113.27	21.08	9.69	8.85	13.4 × 10^3^	987.01	123.47	104.44	−31.52	−1.38	0.71	0.76
	**PM_10_**	0.58	0.48	0.66	0.61	11.28	4.27	2.12	1.99	299.25	59.96	7.27	7.37	−16.30	−2.46	0.61	0.60
	**PM_2.5_**	0.22	0.35	0.64	0.64	6.27	4 × 10^3^	6.75	6.15	139.13	4.48 × 10^7^	115.44	211.02	0.00	−3.2 × 10^5^	0.22	0.43

## Supplementary Materials

The following are available online at https://www.mdpi.com/article/10.3390/s21186228/s1, Spreadsheet S1: Raw data, Hourly data, SLR Results, MLR Results, and MLP Results.
